# Increased Coronary Artery Disease Severity in Black Women Undergoing Coronary Bypass Surgery

**DOI:** 10.1097/MD.0000000000000552

**Published:** 2015-02-20

**Authors:** Jimmy T. Efird, Wesley T. O’Neal, William F. Griffin, Ethan J. Anderson, Stephen W. Davies, Hope Landrine, Jason B. O’Neal, Kristin Y. Shiue, Linda C. Kindell, T. Bruce Ferguson, W. Randolph Chitwood, Alan P. Kypson

**Affiliations:** From the Department of Cardiovascular Sciences (JTE, WFG, KYS, LCK, TBF, WRC, APK), East Carolina Heart Institute; Center for Health Disparities (JTE, HL), Brody School of Medicine, East Carolina University, Greenville; Department of Internal Medicine (WTO), Wake Forest School of Medicine, Winston-Salem; Department of Pharmacology and Toxicology (EJA), Brody School of Medicine, East Carolina University, Greenville, North Carolina; Department of General Surgery (SWD), University of Virginia School of Medicine, Charlottesville, Virginia; and Department of Anesthesia (JBO), Critical Care, and Pain Medicine, Beth Israel Deaconess Medical Center, Harvard Medical School, Boston, Massachusetts.

## Abstract

Race and sex disparities are believed to play an important role in heart disease. The purpose of this study was to examine the association between race, sex, and number of diseased vessels at the time of coronary artery bypass grafting (CABG), and subsequent postoperative outcomes.

The 13,774 patients undergoing first-time, isolated CABG between 1992 and 2011 were included. Trend in the number of diseased vessels between black and white patients, stratified by sex, were analyzed using a Cochran–Armitage trend test. Models were adjusted for age, procedural status (elective vs. nonelective), and payor type (private vs. nonprivate insurance).

Black female CABG patients presented with an increasingly greater number of diseased vessels than white female CABG patients (adjusted *P*_trend_ = 0.0021). A similar trend was not observed between black and white male CABG patients (adjusted *P*_trend_ = 0.18). Black female CABG patients were also more likely to have longer intensive care unit and hospital lengths of stay than other race–sex groups.

Our findings suggest that black female CABG patients have more advanced coronary artery disease than white female CABG patients. Further research is needed to determine the benefit of targeted preventive care and preoperative workup for this high-risk group.

## INTRODUCTION

Currently, 15.4 million (6.4%) Americans have coronary artery disease (CAD), and this number is projected to increase 18% by 2030.^1,2^ Approximately, 397,000 coronary artery bypass grafting (CABG) procedures are performed annually in the United States, with an estimated direct and indirect cost approaching $181 billion.^1^ Although cardiovascular mortality has declined over the last 3 decades, widening disparities in cardiovascular health exist for population subgroups defined by race and sex.^3^

Black women have been observed to use medical services less frequently than other race–sex groups, possibly due to patient–provider mistrust and dissatisfaction with the health care system.^4–7^ Lower health care utilization also has been attributed to provider and health care system biases.^5^ Potentially, this results in delayed diagnosis and advanced cardiovascular disease.

Although previous studies have examined race–sex disparities in cardiovascular disease, this has not been done in the context of CABG surgery.^1,5–9^ The importance of the latter may reflect differences in patient compliance with preoperative management of comorbidities, reluctance of surgeons to operate vis-à-vis quality report cards, perceived lack of postoperative social support, and/or delayed identification of CAD by primary care physicians.^10–12^ Additionally, black women disproportionately receive nonelective CABG for CAD owing to advanced presentation of illness with emergent indications.^13,14^

The purpose of this study was to examine race–sex group disparities in CAD severity, measured by number of disease vessels at the time of presentation for CABG in a large, racially dichotomous, tertiary referral hospital devoted to cardiovascular care. Our findings potentially have important public health implications aimed at improving access to preventive care, earlier screening, intensification of preoperative management, strengthening patient–provider communication and relationships, and ensuring postoperative support.

## METHODS

### Study Design

This was a retrospective analysis of a prospectively maintained database of 13,774 patients undergoing first-time, isolated CABG at the East Carolina Heart Institute between 1992 and 2011 (black men n = 1389, black women n = 990, white men n = 8289, and white women n = 3106). Details of the study database and methodology have been previously described and are summarized below.^11,15–29^ Data collected at the time of surgery included demographics and comorbidities. Only black and white patients were included to minimize the potential for residual confounding (ie, inability to account for confounding, or lack thereof, within each level of the variable), owing to small cell sizes (≤1%) for other races. Patient race was self-reported. The institutional review board at the Brody School of Medicine, East Carolina University approved this study.

### Definitions

Vessels were considered diseased if they had at least 50% stenosis that was confirmed by angiography before surgery. The number of diseased vessels was used as a surrogate indicator of CAD severity.^30^ Chronic obstructive pulmonary disease was classified as “Yes” if at the time of surgery a patient had a forced expiratory volume in 1 second ≤60 and/or was on chronic inhaled or oral bronchodilator therapy.^26,31^ Preoperative heart failure was defined according to the published Society of Thoracic Surgeons (STS) criteria.^29,32^ Additionally, physician documentation or confirmatory medical reports (chest radiographs, consultations, hospital admission notes, medication administration records, outpatient records, physical examinations, and radiology reports) were included. Patients whose symptoms improved after medical therapy or those with stable or asymptomatic compensated failure were not included in this definition. For example, an individual with low ejection fraction without clinical symptoms (edema, paroxysmal nocturnal dyspnea, rales) was not defined as having heart failure. Nondefinitive heart failure diagnoses were ruled out by adjudication, and only definitive cases were included in this study.

Payor status was coded as “private” versus “nonprivate” in order to consolidate different versions of the STS database. Procedure status was defined as elective or nonelective. Age was stratified by Medicare eligibility (<65 years vs. ≥65 years). The absence of an indicated binary variable was defined as the referent group (not shown in tables). Other variables listed in Tables 1 and 2 were coded in accordance with standard STS database definitions.

**TABLE 1:**
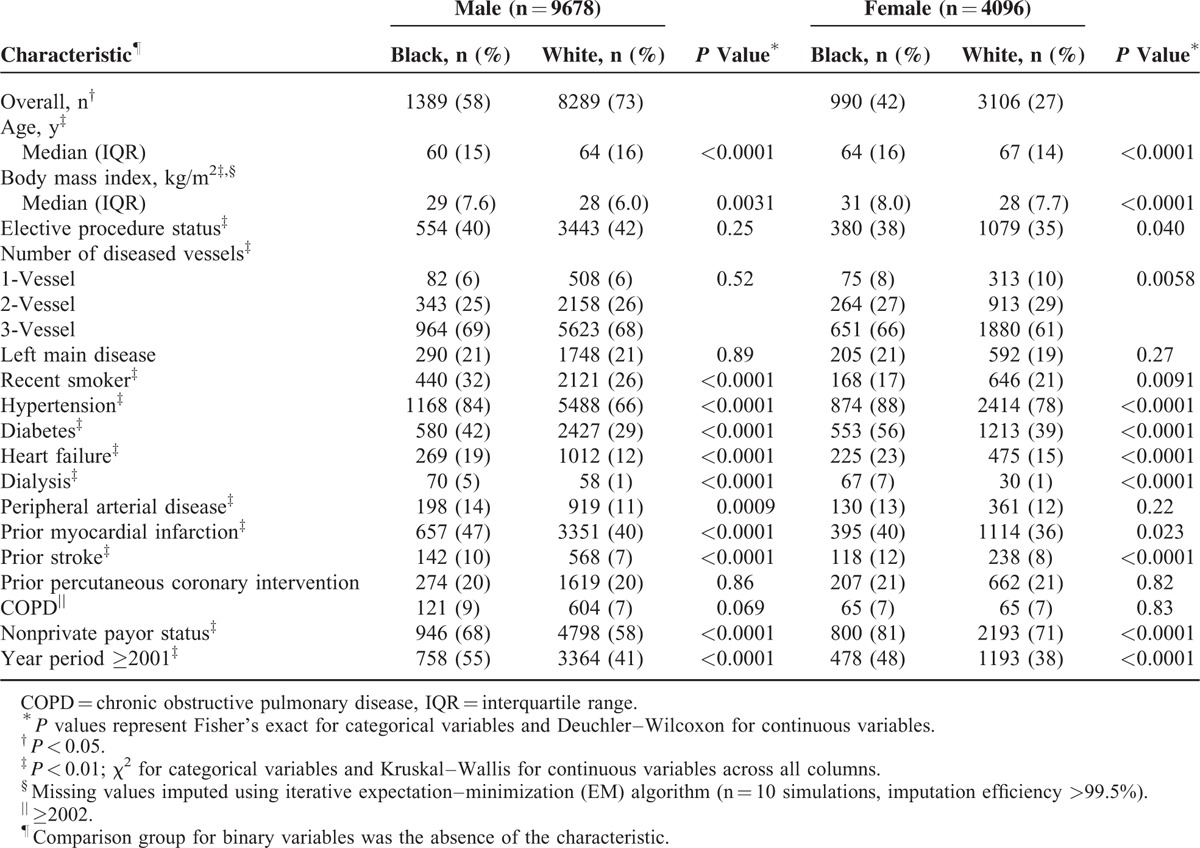
Patient Characteristics (N = 13,774)

**TABLE 2:**
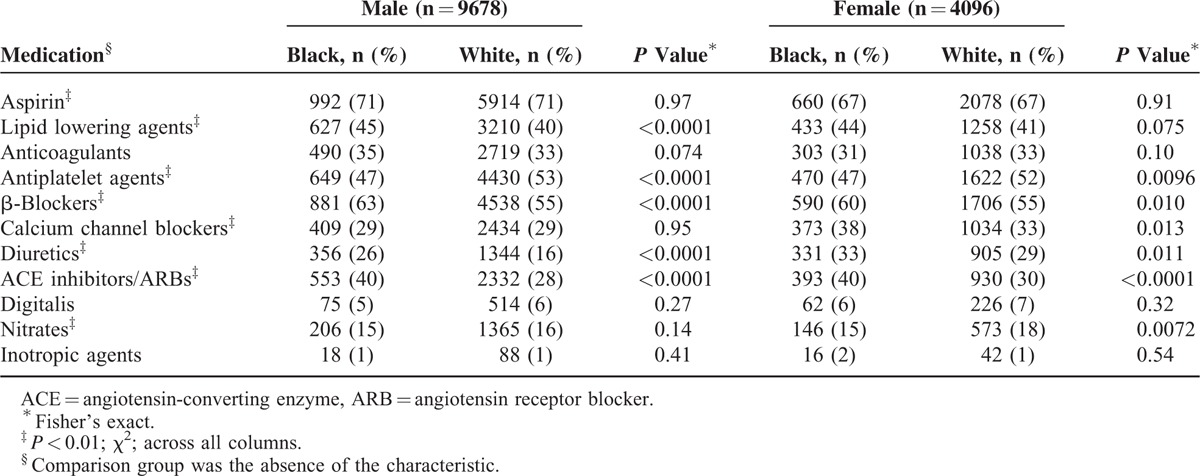
Preoperative Medications

### Data Collection and Follow-Up

The East Carolina Heart Institute is located in eastern North Carolina, a rural region with a high percentage of black patients. As previously described, the institute is the largest stand-alone, tertiary referral facility focusing on cardiovascular care in the state of North Carolina.^11,15–29^ Patients treated at the East Carolina Heart Institute primarily live within a 150-mile radius of the medical center. Data were extracted from the STS Adult Cardiac Surgery Database and the electronic medical records at the affiliated Brody School of Medicine, East Carolina University. The Center for Epidemiology and Outcomes Research at the East Carolina Heart Institute routinely performs validation and assessment for accuracy of the data. Our facility has reported surgical outcomes and data to the STS for over 2 decades. This information is linked to the electronic medical record system in our university-based hospital network. Mismatching of patient data across clinics and follow-up visits was reduced by using multiple logical comparisons.

### Statistical Analysis

Categorical variables were presented as frequency and percentage; continuous variables were presented as the median and interquartile range. Fisher's exact and χ^2^ tests were used to assess statistical significance for categorical variables. Differences in central tendency between or among groups were tested using the Deuchler–Wilcoxon and Kruskal–Wallis procedures. Trend by increasing/decreasing number of diseased vessels was assessed for statistical significance using an exact Cochran–Armitage test. Adjusted *P* values for trend were computed using a multivariable likelihood ratio trend procedure (ordered logit model). Models were stratified by sex and were adjusted for race, age, procedural status, and payor type, based on *a priori* consultation with our cardiothoracic surgeons and supporting literature.^3,8^ The *post hoc* inclusion of other variables (listed in Tables 1 and 2) into the model was performed in a pairwise fashion. Our data contained few missing values (<1%). Missing values were accounted for using the iterative expectation–maximization algorithm.^33–35^ Propensity score matching was not used to adjust for confounding because of “noncollapsibility bias” common to logistic regression based propensity scores.^36^ SAS version 9.3 (SAS Institute Inc, Cary, NC) was used for all analyses, and statistical significance was defined as *P* ≤ 0.05. Access to deidentified data used in our analyses is available upon request and approval by the East Carolina Heart Institution Presentation and Publication Committee.

## RESULTS

Statistically significant race differences (black > white) among both male and female patients were observed for age, body mass index, smoking, hypertension, diabetes, heart failure, dialysis, prior myocardial infarction, prior stroke, nonprivate payor status, year period >2001, antiplatelet agents, β-blockers, diuretics, angiotensin-converting enzyme inhibitors and angiotensin receptor blockers (Tables 1 and 2). Black men (32%) smoked more than white men (26%) (*P* < 0.0001), whereas black women (17%) smoked less than white women (21%) (*P* = 0.0091).

A linear trend was observed for black female CABG patients to present with an increasingly greater number of diseased vessels than white female CABG patients (unadjusted *P*_trend_ = 0.0014 [Table 3], adjusted *P*_trend_ = 0.0021 [not shown in Tables]). The multivariable results did not substantively change with the pairwise addition of other variables listed in Tables 1 and 2, with the exception of diabetes (adjusted *P*_trend_ = 0.026). A similar trend was not observed between black and white male CABG patients (unadjusted *P*_trend_ = 0.31 [Table 3], adjusted *P*_trend_ = 0.18 [not shown in Tables]). Race–sex differences were not observed among privately insured CABG patients.

**TABLE 3:**
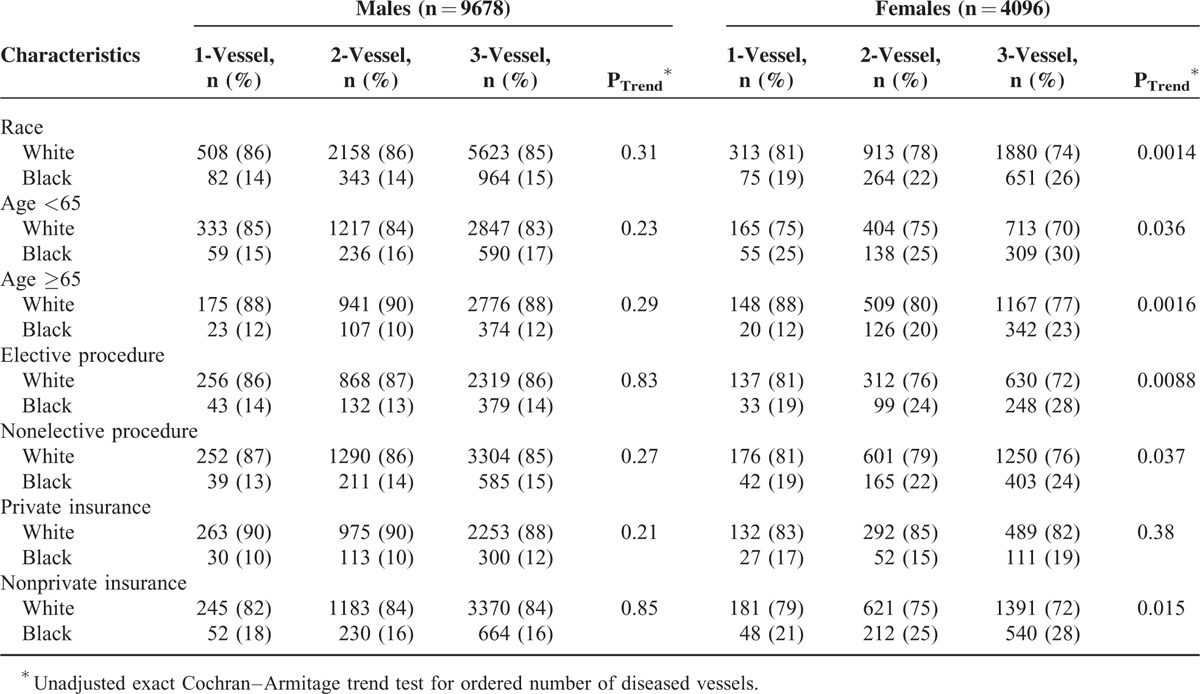
Trend Test for Number of Diseased Vessels

Black women spent a median of 27 hours in the intensive care unit following surgery and stayed in the hospital for a median of 6 days (Table 4). Compared with all other race–sex groups, they experienced longer intensive care unit and hospital lengths of stay (Table 4).

**TABLE 4:**
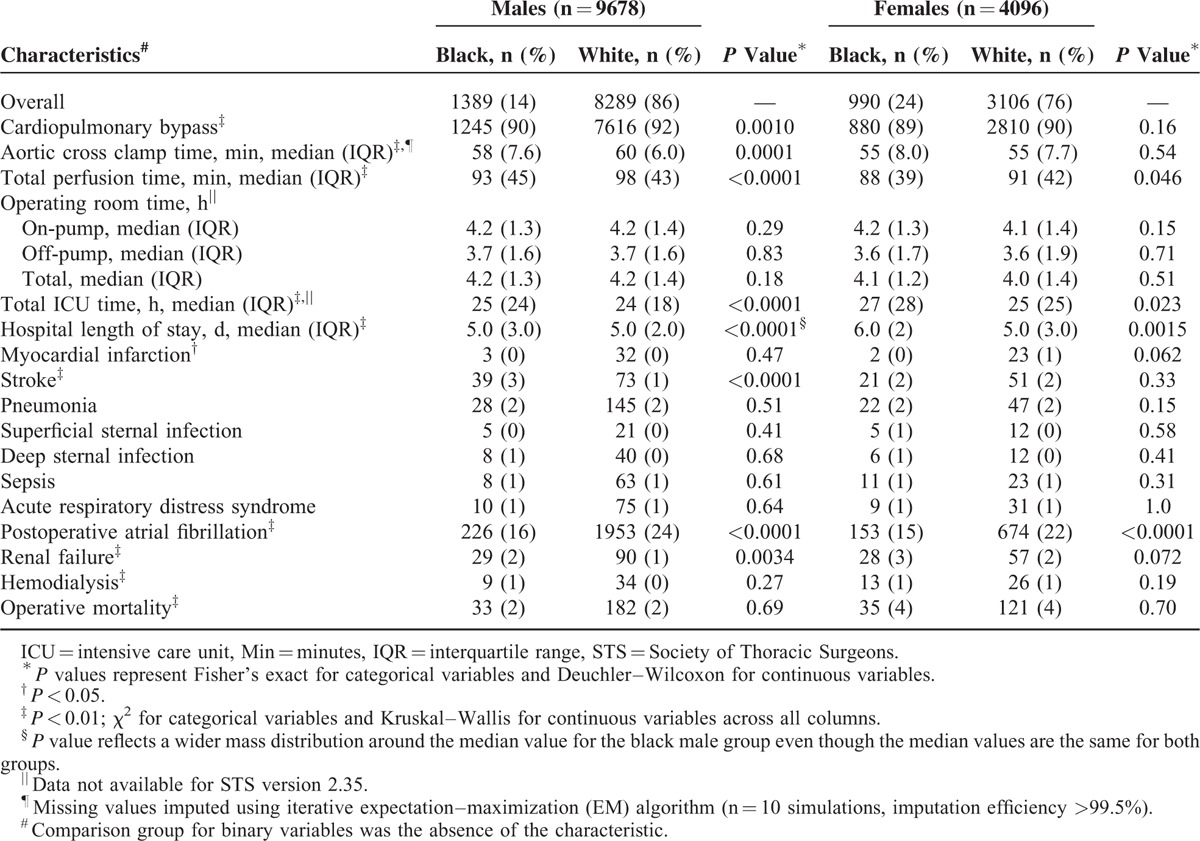
Unadjusted Perioperative Characteristics

## DISCUSSION

In our study, black women undergoing CABG presented with a greater number of diseased vessels than white women. They also experienced longer intensive care unit and hospital lengths of stay than other race–sex groups. The observed disparity in number of diseased vessels may be attributable to several factors including biases at the patient, provider, and health care system levels.

In the general population, CAD differentially affects black women.^1,5–9^ Approximately half of all black women in the United States, 18 years of age or older, have been reported to have varying degrees of cardiovascular disease.^1^ Black women, especially those living in the southeastern region of the United States, have been observed to have the highest national prevalence of obesity, hypertension, and diabetes compared with other groups.^10,37^

Increased levels of proteomic markers (eg, inflammatory, lipoprotein, insulin-resistance promoting adipokines, natriuretic peptides, calcification, and coagulation) for atherosclerosis have been observed among blacks and women, potentially increasing their risk for cardiovascular disease.^38^ Although we were unable to adjust for proteomic markers, our observed findings of increased number of diseased vessels among black female patients at the time of CABG remained statistically significant after controlling for other key predictors of CAD (eg, age, obesity, hypertension, and diabetes).

Several factors may explain our observation that black CABG patients with private insurance (independent of sex) did not present with an increased number of diseased vessels than white CABG patients. Traditionally, private insurance is considered to be a surrogate marker for higher socioeconomic status and decreased CAD incidence.^39,40^ In contrast, low socioeconomic status has been associated with an increase in CAD incidence.^41,42^ Potentially, this is explained by an increased number of jobs, longer work hours, lower salary, and under- or uninsured status, resulting in increased stress and missed medical appointments.^43^ Poor compliance with physician recommendations, patient–provider mistrust, ineffective communication skills, and cultural/religious beliefs also may be important explanatory factors.^5,44^ However, the latter information was not collected in our dataset.

Primary care providers are less likely to refer black female patients to cardiologists and other specialists for diagnostic evaluation.^5,45^ Black communities have fewer primary care physicians than white communities, contributing to the inconsistent follow-up care observed among black patients and the overutilization of emergency departments as their primary source for health care.^43,46^ Health disparities are apparent in the quality of the recipient hospital for patient referrals.^47,48^ An association also has been observed between less affluent geographical regions, higher concentrations of racial/ethnic minorities, and fewer specialists performing fewer procedures.^47^ These regions tend to be associated with worse operative outcomes.^49–51^

Physician bias also may be due to physician quality report cards.^12^ Quality report cards, which are available to the general public, provide risk-adjusted mortality rates for individual surgeons. The unintended consequence of quality report cards is that surgeons may avoid performing high-risk elective CABG procedures involving black women.^12^ Consequently, black women may present with increased CAD severity under nonelective conditions.^13,14^ In the current study, nonelective status was more likely in both blacks and women than in other race–sex groups. Furthermore, difficulties in detecting silent angina among black female patients may have played a role in our findings.

An association between increased postoperative complications/mortality following CABG among blacks and prolonged hospital length of stay has been reported in prior studies.^52–55^ Although an increased rate of postoperative complications and mortality was not observed among black women in our study undergoing CABG, they nonetheless experienced prolonged intensive care unit and hospital lengths of stay. A possible explanation for this discrepancy is that surgeons in our study may have believed that black patients had greater postoperative risk or insufficient home care support than white patients. Furthermore, the higher complication rates reported in previous studies possibly are explained by quality differences among hospitals within a particular setting or region.^53,54^ In contrast, our hospital has consistently received high rankings as a tertiary cardiovascular facility. Further research is needed to determine if earlier hospital discharge with enhanced home care is a safe and cost-effective option for black female CABG patients at our facility.

Our findings provide a potential causative link with population-based studies that have observed an association between race, sex, and cardiovascular outcomes.^56^ For example, a higher incidence of myocardial infarction is observed in the general population for black women compared with white women, and this has consistently held over time periods ranging from 1987 to 2009.^1,8^ Among women, blacks also have been noted to have greater risk for fatal CAD events than whites controlling for age, region of residence, education, and income (hazard ratio = 1.6, 95% CI 1.02–2.6).^8^ This corresponds to a higher operative complication rate and mortality observed among blacks and women following CABG than other groups.^57–59^

### Limitations

Our study did not account for lesion complexity based on pretreatment angiographic criteria, and this potentially resulted in misinterpretation of CAD severity using the 1-, 2-, and 3-vessel categories (eg, 3 focal lesions in the mid portions of the 3 coronary arteries and a left main stem trifurcation lesion may have been similarly classified as 3-vessel disease). Although these criteria are included in the SYNTAX (synergy between percutaneous coronary intervention with TAXUS^®^ (Boston Scientific Corporation, Natick, MA, USA) and cardiac surgery) score, our study period starting in 1992 preceded the development and routine use of this classification system. Education, income, and socioeconomic position were not collected in our database, and these factors may have influenced our findings.^60^ Additionally, a large percentage of our patients live in rural areas with postal box addresses, and thus we were unable to reliably estimate socioeconomic position using zip codes. However, eastern North Carolina is predominately homogenous with respect to socioeconomic status, and it is unlikely that our results would have been substantively affected by adjusting for this variable.

Although we adjusted our analysis for demographic and other potential confounding variables, unmeasured factors could have influenced our results owing to the retrospective, nonrandomized nature of the study. Race was self-reported, and there could have been potential misclassification of this variable. However, the racial make-up of eastern North Carolina is historically dichotomous (black and white), with little miscegenation. Recall and selection bias also are common to retrospective studies.

An increased percentage of black patients in our database received CABG surgery after the year 2001 compared with earlier years. Although this likely reflects institutional efforts to reduce racial disparities in the access and utilization of cardiovascular care, we are unable to rule out other potential clinical explanations for this increase.

Our findings from a single center with a regionally unique population may not generalize to other regions. However, by collecting data from a single health care system, this might have partially controlled for other health care related factors (eg, variation in misclassification of diseased vessels and payor status).

An increased number of diseased vessels presumably are an indicator of delayed diagnosis of CAD. However, confirming this causal link was beyond the scope of the current study.

## CONCLUSION

In our rural and predominately low-socioeconomic region of North Carolina, black women undergoing first-time, isolated CABG presented with a greater number of diseased vessels than white women, and subsequently experienced longer intensive care unit and hospital lengths of stay. Increased attention to patient–provider relationships, communication, and access to health care resources may improve underlying disparities. Public health efforts should also be directed at reversing life course risks factors for CAD including poor diet, inactivity, and stress.

## ACKNOWLEDGMENTS

The authors would like to thank the East Carolina Heart Institute for resources to conduct this study.
